# The sorting of cargo proteins in the plant *trans*-Golgi network

**DOI:** 10.3389/fpls.2022.957995

**Published:** 2022-08-11

**Authors:** Yutaro Shimizu, Tomohiro Uemura

**Affiliations:** ^1^RIKEN Center for Sustainable Resource Science, Wako, Saitama, Japan; ^2^Graduate School of Humanities and Sciences, Ochanomizu University, Bunkyo-ku, Tokyo, Japan

**Keywords:** *trans*-Golgi network, cargo proteins, spatiotemporal, membrane trafficking, RAB GTPase

## Abstract

Membrane trafficking contributes to distinct protein compositions of organelles and is essential for proper organellar maintenance and functions. The *trans*-Golgi network (TGN) acts as a sorting station where various cargo proteins are sorted and directed to post-Golgi compartments, such as the multivesicular body or pre-vacuolar compartment, vacuoles, and plasma membrane. The spatial and temporal segregation of cargo proteins within the TGN, which is mediated with different sets of regulators including small GTPases and cargo adaptors, is a fundamental process in the sorting machinery. Recent studies with powerful imaging technologies have suggested that the TGN possesses spatially distinct subdomains or zones for different trafficking pathways. In this review, we will summarize the spatially and dynamically characteristic features of the plant TGN and their relation to cargo protein trafficking.

## Introduction

**Figure 1:**
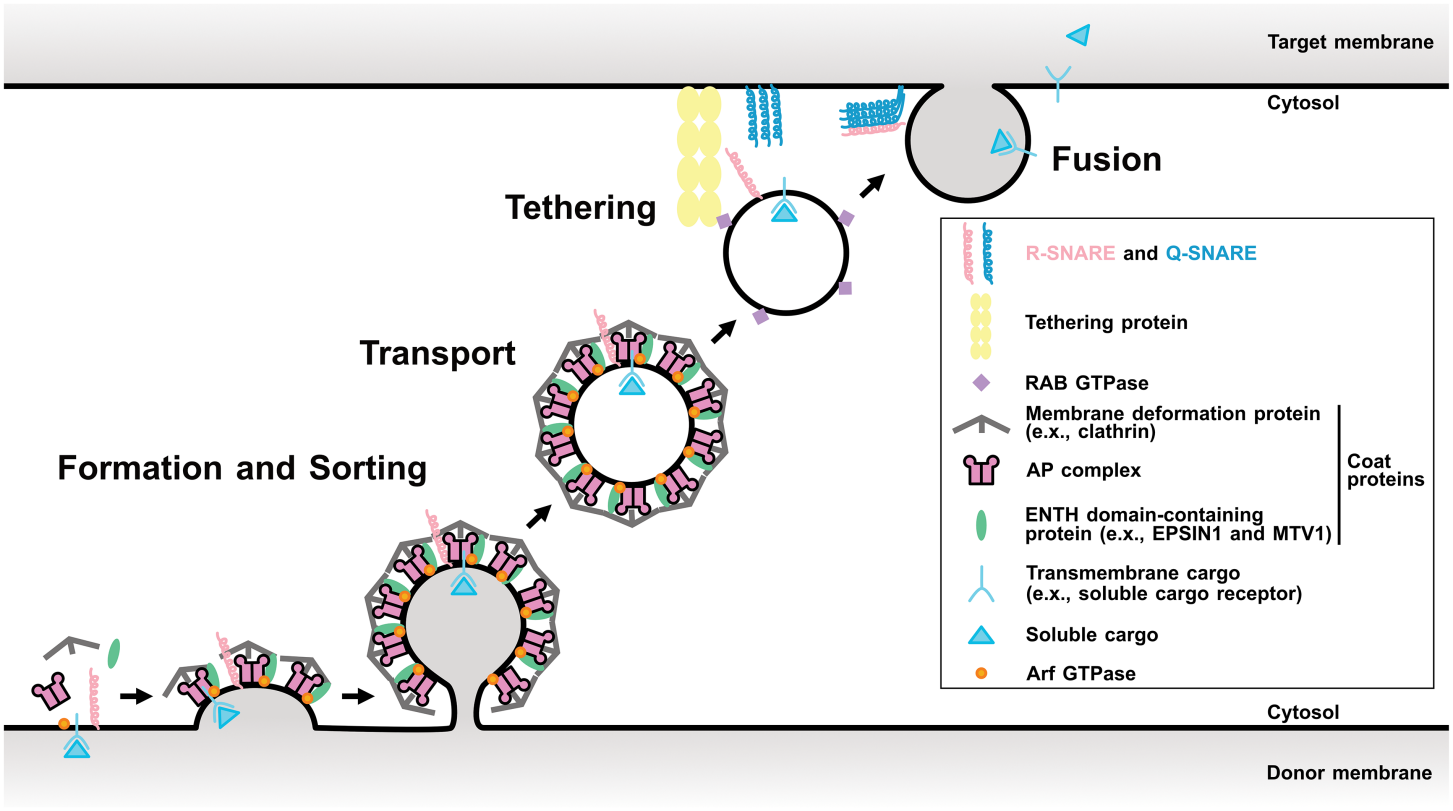
Scheme of a single round of membrane trafficking. General process of membrane trafficking involves the vesicle formation and cargo sorting step, transport, tethering, and finally fusion. Some components of post-Golgi membrane trafficking are showed as representative regulators. Arf GTPase involves in the vesicle formation step. Coat proteins are responsible for the cargo protein sorting and the membrane distortion. RAB GTPase tethers the vesicle by interacting its effectors. SNAREs mediate the membrane fusion between the vesicle and the target membrane. This model is based on the AP-1/clathrin- or AP-2/clathrin-coated vesicles and may be different for other vesicles, such as AP-3-, AP-4-, or AP-5-coated vesicles. AP, adaptor protein; ENTH, epsin N-terminal homology domain; MTV1, MODIFIED TRANSPORT TO THE VACUOLE1.

Membrane trafficking tightly regulates protein localization among organelles and plays a fundamental role in numerous biological processes such as cell growth, development, and stress responses. It is an evolutionarily conserved system among eukaryotes and consists of four fundamental processes: (1) forming transport carriers and sorting cargo proteins on donor organelle membranes; (2) transporting the carriers from the donor to the target organelles; (3) tethering; and (4) fusing them with the target organelle membrane (Fujimoto and Ueda, 2012; Figure 1). These processes are conducted with conserved key regulators and effectors, such as ADP-ribosylation factor/Secretion-associated Ras-related (Arf/Sar) GTPases, coat proteins, RAB GTPases, and soluble N-ethylmaleimide-sensitive factor attachment protein receptors (SNAREs).

First, Arf/Sar GTPases activated by their guanine-nucleotide exchange factor (GEF) promote the recruitment of coat proteins on the donor membrane. Coat proteins play important roles in cargo protein recognition and membrane distortion (Singh and Jürgens, 2018; Arora and Van Damme, 2021; Law et al., 2022). RAB GTPases are involved in various trafficking events, such as tethering (Saito and Ueda, 2009; Minamino and Ueda, 2019). SNAREs mainly function in the last step and can be divided into two groups: target membrane-localized Q-SNAREs and transport carrier-localized R-SNAREs. A specific combination of three Q-SNAREs and one R-SNARE is thought to mediate the fusion between the target membrane and transport carrier (Uemura and Ueda, 2014).

**Figure 2:**
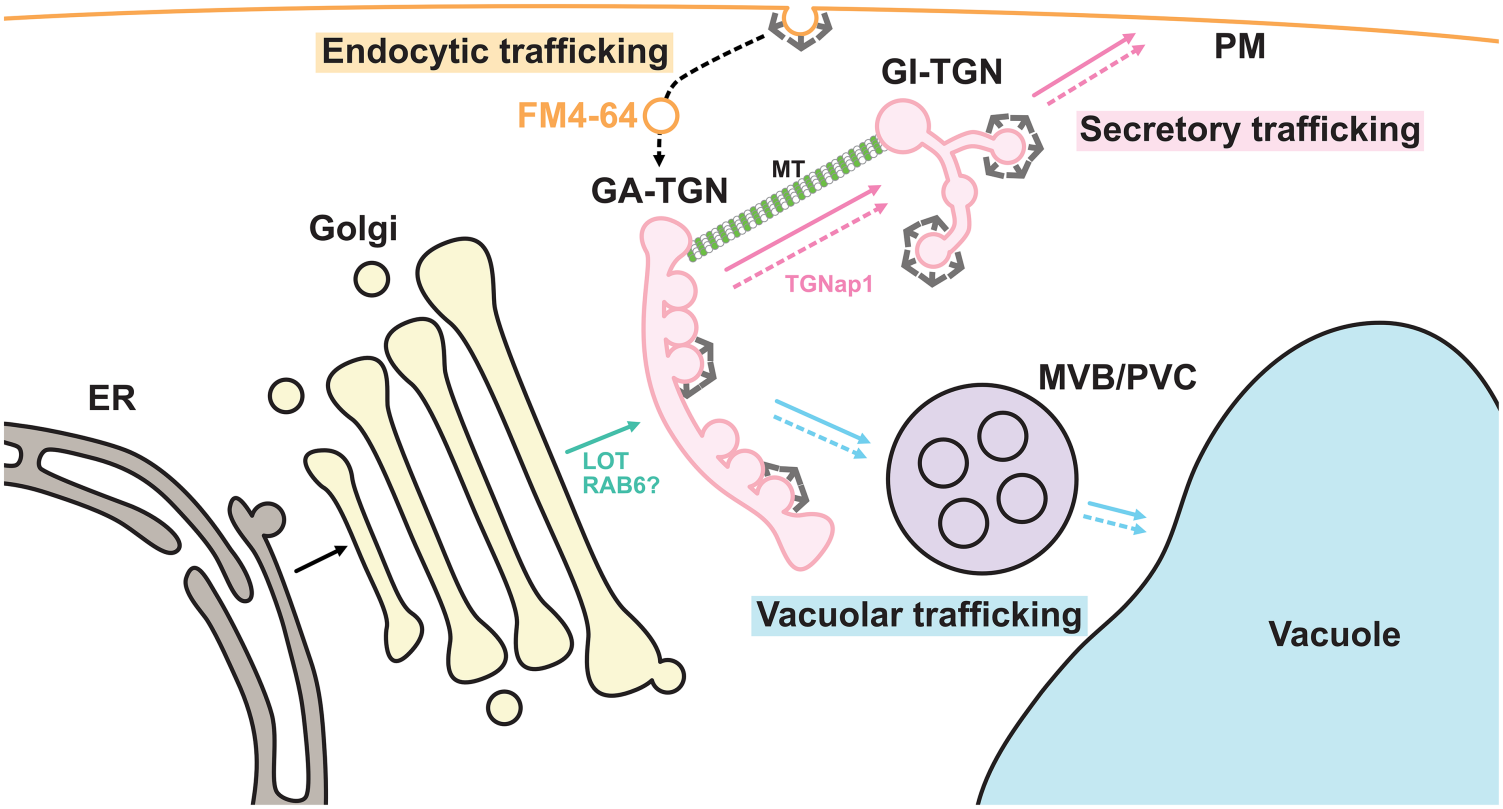
Endomembrane system in plant cells. Plant endomembrane system at least consists of the endoplasmic reticulum (ER), the Golgi apparatus, the *trans*-Golgi network (TGN), the multivesicular body/pre-vacuolar compartment (MVB/PVC), the vacuole and the plasma membrane (PM). The plant TGN can be further classified into the GA-TGN and the GI-TGN. Putative RAB6-GEF LOT plays a critical role in the TGN biogenesis from the TGNs. Microtubule (MT)-binding protein TGNap1 regulates the GI-TGN biogenesis from the Golgi apparatus. The TGN contains cargo proteins derived from the Golgi apparatus, which are synthesized in the ER, and sorts these cargos for delivery to their destinations. In other words, the plant TGN is a branch point in secretory and vacuolar trafficking. In addition, endocytic vesicles labeled with FM4-64 first reach the GA-TGN. Thus, biosynthetic and endocytic trafficking converge at the GA-TGN. The solid arrows and dashed arrows indicate biosynthetic trafficking pathways and endocytic/recycling trafficking pathways, respectively. TGN-bypassing pathways are not shown.

Biosynthetic trafficking starts in the endoplasmic reticulum (ER) where approximately one-third of proteins are synthesized. In normal anterograde membrane trafficking, the *cis* cisterna of the Golgi apparatus receives cargo proteins from the ER, whereas the *trans* cisterna sends them to their destinations, including the plasma membrane (PM) and vacuoles, via the *trans* Golgi network (TGN; Ito and Boutté, 2020; Figure 2). Therefore, the TGN lies in a branch of different trafficking routes and has long been suggested to be the site for cargo sorting. Recent studies have revealed that the TGN harbors spatially segregated functional subdomains or zones for differentially regulated trafficking routes (Heinze et al., 2020; Shimizu et al., 2021). In this review, we will summarize and discuss the recent discoveries that have been made on TGN-mediated trafficking.

## The *trans*-Golgi network

The TGN is generally recognized as a membranous structure at the *trans*-side of the Golgi apparatus (Griffiths and Simons, 1986; Ito and Boutté, 2020), which partially corresponds to the compartment previously proposed as the Golgi-associated structure that is a part of the ER and forms Lysosomes (GERL) in the 1970s or termed the partially coated reticulum (PCR) in 1980s (Marty, 1978; Harris and Oparka, 1983; Pesacreta and Lucas, 1984; Staehelin et al., 1990). Morphologically distinct types of vesicles or multiple coat proteins have been found in the TGN, indicating that it is an important site for sorting cargo proteins with different destinations (Griffiths and Simons, 1986; Singh and Jürgens, 2018). Cargo proteins are thought to be transported from the Golgi to the TGN by the process of “cisternal maturation,” which has been well-studied in budding yeast (Glick and Nakano, 2009). According to this model, the *trans*-most Golgi cisterna is the cargo carrier and matures into the TGN without packing cargo molecules into the nascent transport carrier. Therefore, the TGN plays an essential role in sorting mixtures of biosynthetic cargo proteins with different destinations, which are derived from the Golgi apparatus. In partial support of this hypothesis, a knockout mutant of a Golgi-localized putative GEF for RAB6 (termed the “loss of TGN,” or LOT) was found to impair the biogenesis of the TGN, while the Golgi apparatus became over-stacked and elongated in *Arabidopsis* (Jia et al., 2018, 2019).

Interestingly, the plant TGN is a unique compartment that also functions as an early endosome where endocytosed molecules are first delivered. In a canonical model based on mammalian studies, endocytosed materials first reached the early endosome, which was physically separated from the TGN. They were then recycled back to the PM or redirected to lysosomes (Rohn et al., 2000; Barlow and Dacks, 2018). In plant cells, however, the lipophilic fluorescent dye FM4-64, which is gradually internalized from the PM by the endocytic machinery, first reached the TGN before other endomembrane organelles (Dettmer et al., 2006; Lam et al., 2007; Chow et al., 2008). Endocytic cargo proteins, such as BRASSINOSTEROID INSENSITIVE1 and REQUIRES HIGH BORON1, have also been reported to pass through the TGN (Viotti et al., 2010). A recent study involving the budding yeast *Saccharomyces cerevisiae* further reported that endocytosed FM4-64 first colocalized with the TGN marker (Day et al., 2018). These findings suggest that the TGN functions as an early endosome in plants and budding yeast and plays important roles not only in biosynthetic trafficking pathways, but also in endocytic trafficking pathways (Figure 2).

## Spatiotemporal characteristics of the TGN

**Figure 3:**
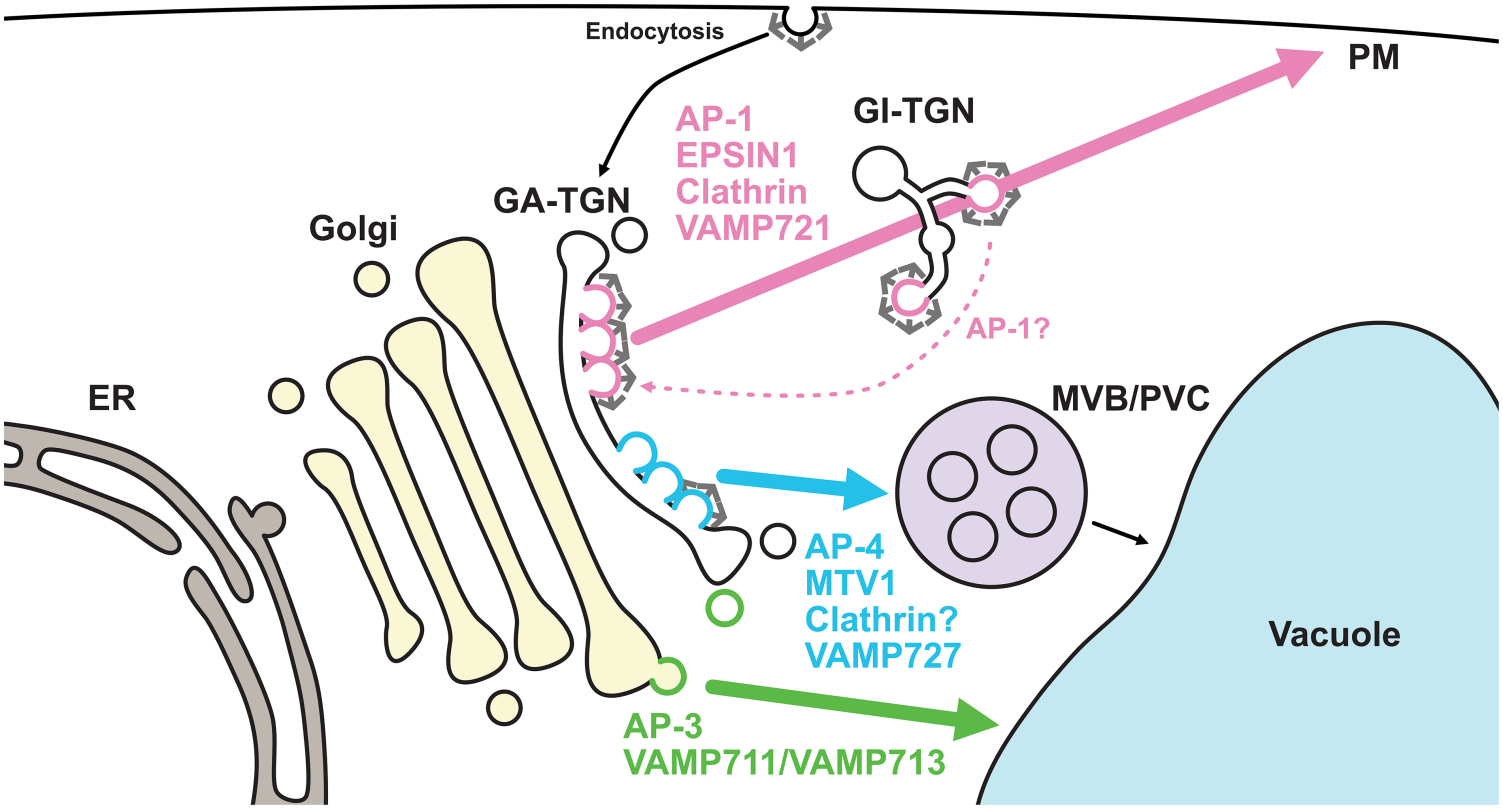
Golgi- and TGN-localized coat proteins and R-SNAREs regulate distinct pathways. TGN-localized coat proteins and R-SNAREs exhibit spatially distinct distributions. AP-1 (coat), EPSIN1 (coat), clathrin (coat), and VAMP721 (R-SNARE) comprise a subdomain or zone of the GA-TGN, which is involved in secretory trafficking. On the other hand, AP-4 (coat), MTV1 (coat), and VAMP727 (R-SNARE) comprise another subdomain or zone on the same GA-TGN, which is involved in vacuolar trafficking via the MVB/PVC. It remains controversial whether AP-4-mediated trafficking requires clathrin. The former subdomain or zone matures into the GI-TGN. The GI-TGN produces non-coated secretory and clathrin-coated vesicles. AP-1/clathrin-coated vesicles may play a role in retrograde trafficking from the GI-TGN. AP-3-coated vesicles may be formed at the *trans*-cisterna of the Golgi rather than the TGN and transport vacuolar cargo proteins, such as VAMP711 and VAMP713, to vacuoles independently of MVB/PVC. ER, endoplasmic reticulum; GA-TGN, Golgi-associated TGN; GI-TGN, Golgi-independent TGN; MVB/PVC, multivesicular body/pre-vacuolar compartment; PM, plasma membrane; AP, adaptor protein; MTV1, MODIFIED TRANSPORT TO THE VACUOLE1; VAMP, vesicle-associated membrane protein.

Although the concept of the TGN as the sorting platform in post-Golgi membrane trafficking is widely accepted, the mechanisms by which the TGN directs multiple sorting events remain unknown. To do this, the TGN must pack cargos with different destinations into distinct vesicles and/or sort them into different subdomains or zones. Recent studies have revealed that the TGN harbors at least two distinct domains or zones for different trafficking pathways (Heinze et al., 2020; Shimizu et al., 2021; Figure 3).

In a branch of the post-Golgi trafficking pathways, R-SNAREs with different destinations, VAMP721 and VAMP727, localize to the TGN. VAMP721 functions in the trafficking to the PM and cell plate, whereas VAMP727 mainly functions in the trafficking in the vacuolar pathway (Ebine et al., 2008; Kwon et al., 2008; Zhang et al., 2011; El Kasmi et al., 2013). Recently, we used super-resolution confocal live imaging microscopy (SCLIM) to reveal that VAMP721 and VAMP727 were spatially segregated as subdomains or zones, but not vesicles, within a single TGN (Shimizu et al., 2021). The VAMP721-localized subdomains and VAMP727-localized subdomains were proposed as the “secretory-trafficking zone” and “vacuolar-trafficking zone,” respectively. A similar segregation pattern was also seen in multiple coat proteins. The localizations of TGN-resident coat proteins, including adaptor protein complex 1 (AP-1), AP-4, EPSIN1, MODIFIED TRANSPORT TO THE VACUOLE1 (MTV1), and clathrin, have been investigated by SCLIM or high-resolution Airyscan imaging (Heinze et al., 2020; Shimizu et al., 2021). The comprehensive analyses revealed that the secretory-trafficking zone was enriched with VAMP721, AP-1, EPSIN1, and clathrin, whereas the vacuolar-trafficking zone was enriched with VAMP727, AP-4, and MTV1. Thus, secretory and vacuolar cargo proteins may be sorted via the spatially distinct zones within a single TGN.

In addition to its sub-organellar features, the plant TGN is classifiable at the spatiotemporal level. Fast live imaging by spinning disk confocal microscopy and 3D reconstruction by electron tomography have revealed that the plant TGN can dissociate from or associate with the Golgi apparatus (Staehelin and Kang, 2008; Viotti et al., 2010; Kang et al., 2011; Uemura et al., 2014). The canonical and non-canonical modes of the TGN have been termed as the Golgi-associated TGN (GA-TGN) and Golgi-independent TGN (free-TGN/GI-TGN), respectively. The detachment of the GI-TGN from the GA-TGN requires TGNap1, which likely functions as a linker between the TGN and microtubules (Renna et al., 2018). At the component level, the GA-TGN harbors VAMP721, or its close homolog VAMP722, and VAMP727. In contrast to the GA-TGN, the GI-TGN is enriched with VAMP721 and/or VAMP722, but not VAMP727, suggesting that the GI-TGN predominantly mediates secretory trafficking to the PM (Uemura et al., 2019). In the earlier stages (approximately 5 min after uptake), endocytosed FM4-64 colocalizes with the GA-TGN, AP-1, and AP-4, but not the GI-TGN (Uemura et al., 2019; Shimizu et al., 2021). These results highlight the importance of the GA-TGN as a sorting hub for secretory, vacuolar, and endocytic trafficking. The GI-TGN further harbors both AP-1/clathrin-coated and secretory vesicles, and the ratio of secretory vesicles to clathrin-coated vesicles can vary, even within single cells (Kang et al., 2011; Shimizu et al., 2021).

## Coat proteins at the TGN

Heterotetrameric AP complexes and clathrin are well-characterized coat proteins and function in post-Golgi membrane trafficking. Five AP complexes (AP-1–AP-5) are derived from a common ancestral complex and are thought to have been present in the last eukaryotic common ancestor (Hirst et al., 2011; Dacks and Robinson, 2017). Hence, they are evolutionarily conserved in eukaryotes. AP complexes typically consist of two large subunits (β and α/γ/δ/ε/ζ), a medium subunit (μ), and a small subunit (σ). They sort membrane-bound or transmembrane cargo proteins by recognizing and binding to sorting motifs or signals, such as the tyrosine motif, dileucine motif, and ubiquitin, which are present in the cytoplasmic domains of cargo proteins (Arora and Van Damme, 2021; Law et al., 2022). Various AP complexes interact with clathrin to form clathrin-coated vesicles. Well-established examples include AP-1/clathrin-coated vesicles (Park et al., 2013) and AP-2/clathrin-coated vesicles (Di Rubbo et al., 2013; Kim et al., 2013; Yamaoka et al., 2013). Each of the AP complexes functions in distinct trafficking pathways in eukaryotes. In *Arabidopsis*, both AP-1 and AP-4 function in the TGN (Park et al., 2013; Teh et al., 2013; Wang et al., 2013; Fuji et al., 2016).

AP-1 is essential for survival as *ap1γ1 ap1γ2* double mutants cannot be obtained (Wang et al., 2014). *ap1μ2* (major isoform of AP-1 μ subunits) mutants have retarded growth and are unable to produce progenies (Park et al., 2013). In contrast to AP-4 as described below, AP-1 seems to play roles in a wide range of trafficking pathways and localizes to the TGN secretory-trafficking zone with VAMP721. For example, secretory invertase and GFP are not properly secreted into the extracellular medium, whereas the vacuolar proteins sporamin and βFructosidase4 are unprocessed in *ap1m2-1/hap13-1* protoplasts in *Arabidopsis*. Furthermore, PIN-FORMED2 (PIN2) polar recycling from the brefeldin A (BFA) compartment to the PM or vacuoles is compromised in *ap1m2-1/hap13-1* seedlings. BRASSINOSTEROID INSENSITIVE1 recycling from the BFA compartment to the PM is also compromised in *ap1m2-1/hap13-1* seedlings (Park et al., 2013; Wang et al., 2013). It has also been reported that the AP-1 μ subunit can bind transmembrane receptors for vacuolar soluble proteins known as vacuolar sorting receptors (VSRs; Park et al., 2013; Gershlick et al., 2014; Nishimura et al., 2016). In addition, the trafficking of cytokinesis-specific SNARE KNOLLE/SYP111 to the cell plate is severely impaired, leading to incomplete cytokinesis (Park et al., 2013; Teh et al., 2013). Severe growth defects in *ap1m2-1/hap13-1* mutants can be rescued by expressing functional AP1M2 with the KNOLLE/SYP111 promoter as well as AP1M2 promoter (Teh et al., 2013; Shimada et al., 2018). Interestingly, outermost integument cells of the pKNOLLE:AP1M2-GFP-rescued *ap1m2-1/hap13-1* seed lack AP1M2 expression and exhibit reduced mucilage extrusion and its abnormal accumulation in vacuoles. This suggests that AP-1 is also involved in the secretory trafficking of macromolecules and cargo proteins (Shimada et al., 2018). Furthermore, the disruption of AP-1 function has been found to reduce the PM association of AP-2, clathrin, and the clathrin adaptor TPLATE complex. This impairs clathrin-mediated endocytosis and vice versa (Yan et al., 2021). Therefore, AP-1/clathrin-mediated trafficking from the TGN and AP-2/clathrin-mediated trafficking from the PM couple by unknown mechanisms. AP-1 is therefore involved in the secretory, vacuolar, and recycling trafficking pathways, although it remains unclear how AP-1 can manage such complicated sorting events.

Furthermore, we have assessed the role of AP-1 from the spatiotemporal perspective. SCLIM observations have revealed that AP-1- and clathrin-labeled compartments leave the GA-TGN with SYNTAXIN OF PLANTS61 (SYP61) as components of the GI-TGN, while AP-4 seems to stay with the GA-TGN (Shimizu et al., 2021). Electron tomography has further revealed that the GI-TGN harbors clathrin-coated and secretory vesicles (Kang et al., 2011). These findings suggest that AP-1/clathrin-coated vesicles may function in the GI-TGN. Intriguingly, Yan *et al*. recently reported that the TGN-resident SNAREs SYP41 and SYP61, but not VHAa1, were significantly dissociated from the TGN and mislocalized to the PM in *ap1m2/hap13* root cells. This suggests that AP-1 is responsible for maintaining the functional integrity of the TGN (Yan et al., 2021). In support of this hypothesis, *ap1m2-1/hap13-1* mutants have been found to exhibit abnormal morphologies in the Golgi and TGN (Park et al., 2013). It has also been reported that SYP4s and SYP61 regulate both secretory and vacuolar trafficking (Bassham et al., 2000; Uemura et al., 2012; Hachez et al., 2014; Lu et al., 2020). These findings indicate the possibility that AP-1/clathrin-coated vesicles may deliver TGN-resident proteins and missorted vacuolar proteins from the GI-TGN to the GA-TGN or Golgi apparatus. Recent studies involving budding yeast have further reported that AP-1 plays a role in the retrograde transport of cargo proteins from mature to young Golgi cisternae (Casler et al., 2019, 2022; Casler and Glick, 2020). It should be noted that, although the mechanisms are unknown, the quantity of GA-TGN-localized proteins seems to be maintained at a relatively stable level, as the fluorescence of TGN markers, including SYP43 and SYP61, seems to be recovered around the GA-TGN region after producing the GI-TGN (Uemura et al., 2014; Shimizu et al., 2021).

In a forward genetic screen in *Arabidopsis*, AP-4 components were reported to be responsible for the vacuolar sorting of GFP-CT24, which is an artificial cargo protein with β-conglycinin-derived vacuolar targeting signals (Fuji et al., 2007, 2016). The tonoplast proteins molybdate transporter 2 and natural resistance macrophage protein 3 and 4 were missorted to the PM in *ap4m* mesophyll protoplasts (Müdsam et al., 2018). In addition, the μ subunit of AP-4 and that of AP-1 mentioned above have been found to bind to the tyrosine motifs of VSR1 and VSR2 (Gershlick et al., 2014; Fuji et al., 2016). Given that the putative cargo VAMP727 mainly functions in the multivesicular body (MVB; Ebine et al., 2008) and that the VSR-mediated trafficking pathway relies on the MVB to deliver cargo proteins to vacuoles, AP-4 likely functions as a gatekeeper in the pathway from the TGN to MVB, although it remains controversial where VSR itself travels (Ivanov and Robinson, 2020). MTV1, an accessory protein of AP-4, has also been identified as a responsible gene for the vacuolar trafficking of the artificial cargo protein VAC2 (Sauer et al., 2013). Thus, genetic and biochemical analyses support the role of AP-4 in vacuolar trafficking. The question remains as to whether plant AP-4 is a component of clathrin-coated vesicles *in vivo*. Coimmunoprecipitation assays have shown that the μ subunit of AP-4 immunoprecipitates clathrin heavy chains (Fuji et al., 2016; Shimizu et al., 2021). However, SCLIM observations of *Arabidopsis* root cells have shown that AP-4 is segregated from clathrin as opposed to AP-1 (Shimizu et al., 2021). Recently, Dahhan *et al*. characterized the proteome of purified plant clathrin-coated vesicles and found that AP-4 was enriched in the clathrin-coated vesicle fraction (Dahhan et al., 2022). Thus, it remains controversial whether clathrin is required for plant AP-4-mediated trafficking *in vivo*. The way in which AP-4 localizes to the TGN also remains unknown. Although the ARF-GEFs BIG1–BIG4 and ARF1 have been shown to recruit AP-1 to the TGN membrane (Richter et al., 2014; Singh et al., 2018), it is unclear which ARF-GEFs and ARFs are required for localizing AP-4 to the TGN.

Several vacuolar proteins are transported to vacuoles independently of MVB-mediated pathways. A striking example is the AP-3-dependent pathway. The vacuolar membrane targeting of VAMP711/VAMP713 and PROTEIN S-ACYL TRANSFERASE10 is mediated by AP-3 independent of the MVB-pathway requiring RAB5 (Ebine et al., 2014; Feng et al., 2018). Studies in mammalian models have reported that AP-3 sorts lysosomal cargo proteins in the TGN and endosomes (Peden et al., 2004; Huang et al., 2019). However, it remains unclear whether plant AP-3 constitutes a distinct zone within the TGN like AP-1 and AP-4 since the localization of AP-3 is not well defined. Fluorescent protein-labeled AP-3 exhibits a relatively high degree of colocalization with the Golgi markers WAVE22/SYP32 and TGN marker VHAa1 (Feng et al., 2017). Studies on mammalian cells and the biochemical interactions of plant AP-3 with clathrin (Zwiewka et al., 2011) may confuse the precise interpretation of AP-3 localization in plant cells. Intriguingly, PROTEIN S-ACYL TRANSFERASE10 seems to be retained in the Golgi but not TGN in *ap3δ* mutants, suggesting that AP-3 sorts vacuolar cargo proteins at the Golgi apparatus rather than the TGN (Feng et al., 2018). Further investigations on AP-3 localization will contribute to a better understanding why and how vacuolar trafficking is regulated by different components.

## Complicated secretory trafficking from the TGN

The studies on secretory-trafficking and vacuolar-trafficking zones marked with VAMP721 and VAMP727, respectively, appear to be oversimplified. For example, there are two different routes from the TGN to the PM. The most striking discovery is that the polar recycling of PIN1 to the basal PM is regulated by ARF-GEF GNOM, whereas the recycling of apical, lateral, and nonpolar PM proteins is regulated by RABA2a (Geldner et al., 2003; Li et al., 2017). In these studies, BFA and Endosidin16, chemical inhibitors of membrane trafficking, are used as a powerful tool to dissect the different trafficking routes. BFA binds between the GDP-bound ARF/SAR and its GEF, thereby blocking a subset of secretion from the Golgi and/or TGN and early secretory trafficking from the ER to the Golgi (Jackson and Casanova, 2000). Endosidin16 compromises RABA2A-mediated trafficking from the TGN (Li et al., 2017). It should be noted that the establishment and maintenance of PIN polarity are coordinated by endocytosis and polar recycling (Geldner et al., 2003; Men et al., 2008; Kitakura et al., 2011; Glanc et al., 2018), as well as the *de novo* delivery of PIN2 (Wattelet-Boyer et al., 2016).

Rodriguez-Furlan *et al*. recently reported that newly synthesized INFLORESCENCE AND ROOT APICES RECEPTOR KINASE (IRK) and KINASE ON THE INSIDE (KOIN) are sorted at the TGN by different machineries and transported to the opposite sides (i.e., outer and inner sides, respectively) of the root endodermal PM. These different *de novo* pathways have also been demonstrated using BFA and Endosidin16 in combination with or without the protein synthesis inhibitor cycloheximide. Trafficking of IRK is compromised with BFA but not Endosidin16, whereas that of KOIN is sensitive to Endosidin16 but not BFA (Rodriguez-Furlan et al., 2022). Interestingly, the PM targeting of both IRK and KOIN is also compromised with Endosidin2 (Rodriguez-Furlan et al., 2022). Endosidin2 inhibits exocytosis by binding the EXOCYST subunit EXO70A1 that plays important roles in targeted secretion and tethering at the PM (Zhang et al., 2016). These results suggest that IRK and KOIN are sorted into distinct trafficking pathways at the TGN; however, they are regulated by EXOCYST-mediated tethering mechanisms on the way to and/or at the PM. As some cargo proteins are exported from the TGN by different machinery, so some cargo proteins are regulated by distinct tethering and SNARE proteins on the target membrane. Plasma membrane-localized Q-SNAREs SYP121/ PEN1 (PENETRATION1) and SYP122 have partially redundant functions, since *syp121 syp122* double mutants display growth defects that are not observed in either single mutant (Collins et al., 2003; Assaad et al., 2004; Zhang et al., 2007). However, mass spectrometry analysis using the *syp121* and *syp122* mutants revealed that SYP121 and SYP122 mediate the secretion of partially overlapping but distinct sets of distinct cargo proteins (Waghmare et al., 2018). Other studies also show that K^+^ channels bind selectively to SYP121 but not SYP122 (Honsbein et al., 2009, 2011; Grefen et al., 2010). The RABA2a-dependent and RABE1-EXOCYST-dependent secretory pathways, which coexist in *Arabidopsis*, have been also proposed (Pang et al., 2022). Given these studies, the secretory-trafficking zone of the TGN may further be classified into the sub-zone level or the single-vesicle level.

## Discussion

It is now clear that the plant TGN is the central hub of secretory, vacuolar, and endocytic trafficking. Accumulating evidence suggests that the TGN can be divided into subdomains or zones where distinct trafficking events are executed. Such subdomain/zone-like segregation has been seen in various TGN-localized proteins including coat proteins, SNAREs, and tethering factors (Bassham et al., 2000; Chow et al., 2008; Gendre et al., 2011, 2013; Boutté et al., 2013; Wattelet-Boyer et al., 2016; Ravikumar et al., 2018; Renna et al., 2018; Heinze et al., 2020; Shimizu et al., 2021). However, it remains poorly understood how these subdomains or zones are organized. Lipid composition of the organelle membrane can regulate protein localization and thus plays an important role in endomembrane trafficking and diverse cellular functions (Bigay and Antonny, 2012; Holthuis and Menon, 2014; Boutté and Jaillais, 2020). Interestingly, it has been reported that α-hydroxylated-very long chain fatty acid-containing sphingolipids are specifically enriched in the immunoisolated SYP61-, but not RABA2a-, localized TGN (Wattelet-Boyer et al., 2016). Pharmacological reduction of very long chain fatty acid-containing sphingolipids disturbs the polar delivery of PIN2 (Wattelet-Boyer et al., 2016). Furthermore, α-hydroxylated-very long chain fatty acids are involved in regulating the quantity of phosphatidylinositol 4-phosphate (PI4P) in the TGN (Ito et al., 2021). In plant cells, PI4P mainly accumulates at the PM and to a lesser extent at the TGN. TGN-localized phosphatidylinositol 4-kinases *pi4kβ1 pi4kβ2* double mutants have the abnormal TGN with highly variable sizes of secretory vesicles and exhibit pleiotropic growth defects (Preuss et al., 2006; Kang et al., 2011; Sašek et al., 2014; Antignani et al., 2015; Lin et al., 2019). In *Arabidopsis*, ARF-GAP VASCULAR NETWORK DEFECTIVE3 and RABA4b effector PLANT U-BOX13 have been shown to bind PI4P and to localize to the TGN (Koizumi et al., 2005; Naramoto et al., 2009; Antignani et al., 2015). It has also been reported that the mammalian AP-1 complex binds PI4P (Wang et al., 2003). Therefore, distinct lipid environments likely serve as platforms for organizing subdomains or zones in the TGN, which will be investigated in the near future. To better understand sub-organellar features, cutting-edge imaging technologies are essential. For example, correlative light and electron microscopy will fill the gaps in the current knowledge on morphological features and fluorescence-based protein localization (Wang and Kang, 2020). In addition, to better understand how cargo proteins are sorted into specific TGN subdomains/zones, we should evaluate the association between the organization of trafficking zones and cargo flow. Because the passage of cargo proteins is transient, it is necessary to establish a pulse-chase-type experimental system. Several methods have been proposed and successfully applied in other organisms, such as retention using a selective hooks system in mammalian cultured cells (Boncompain et al., 2012) and a temperature-controlled system in budding yeast (Kurokawa et al., 2014). However, such systems have not yet been established in plant cells. Given that membrane trafficking appears to be intricately regulated during developmental processes and environmental changes, it is important to investigate how cargo proteins are transported in various conditions.

## Author contributions

YS and TU wrote and edited the manuscript and prepared the figure. All authors contributed to the article and approved the submitted version.

## Funding

This work was supported by Grants-in-Aid for Scientific Research from the Ministry of Education, Culture, Sports, Science and Technology (grant number: 18H04857 to TU), JST (grant number: JPMJCR20E5 to TU), Grant for Basic Science Research Project from Sumitomo Foundation (to TU), and JSPS Research Fellowship for Young Scientists (to YS).

## Conflict of interest

The authors declare that the research was conducted in the absence of any commercial or financial relationships that could be construed as a potential conflict of interest.

## Publisher’s note

All claims expressed in this article are solely those of the authors and do not necessarily represent those of their affiliated organizations, or those of the publisher, the editors and the reviewers. Any product that may be evaluated in this article, or claim that may be made by its manufacturer, is not guaranteed or endorsed by the publisher.

## References

[ref1] Antignani V. Klocko A. L. Bak G. Chandrasekaran S. D. Dunivin T. Nielsen E. (2015). Recruitment of PLANT U-BOX13 and the PI4Kβ1/β2 phosphatidylinositol-4 kinases by the small GTPase RabA4B plays important roles during salicylic acid-mediated plant defense signaling in Arabidopsis. Plant Cell 27, 243–261. doi: 10.1105/tpc.114.134262, PMID: 25634989 PMC4330583

[ref2] Arora D. Van Damme D. V. (2021). Motif-based endomembrane trafficking. Plant Physiol. 186, 221–238. doi: 10.1093/plphys/kiab077, PMID: 33605419 PMC8154067

[ref3] Assaad F. F. Qiu J. L. Youngs H. Ehrhardt D. Zimmerli L. Kalde M.. (2004). The PEN1 syntaxin defines a novel cellular compartment upon fungal attack and is required for the timely assembly of papillae. Mol. Biol. Cell 15, 5118–5129. doi: 10.1091/mbc.e04-02-0140, PMID: 15342780 PMC524786

[ref4] Barlow L. D. Dacks J. B. (2018). Seeing the endomembrane system for the trees: evolutionary analysis highlights the importance of plants as models for eukaryotic membrane-trafficking. Semin. Cell Dev. Biol. 80, 142–152. doi: 10.1016/j.semcdb.2017.09.027, PMID: 28939036

[ref5] Bassham D. C. Sanderfoot A. A. Kovaleva V. Zheng H. Raikhel N. V. (2000). AtVPS45 complex formation at the trans-Golgi network. Mol. Biol. Cell 11, 2251–2265. doi: 10.1091/mbc.11.7.2251, PMID: 10888666 PMC14917

[ref6] Bigay J. Antonny B. (2012). Curvature, lipid packing, and electrostatics of membrane organelles: defining cellular territories in determining specificity. Dev. Cell 23, 886–895. doi: 10.1016/j.devcel.2012.10.009, PMID: 23153485

[ref7] Boncompain G. Divoux S. Gareil N. de Forges H. Lescure A. Latreche L.. (2012). Synchronization of secretory protein traffic in populations of cells. Nat. Methods 9, 493–498. doi: 10.1038/nmeth.1928, PMID: 22406856

[ref8] Boutté Y. Jaillais Y. (2020). Metabolic cellular communications: feedback mechanisms between membrane lipid homeostasis and plant development. Dev. Cell 54, 171–182. doi: 10.1016/j.devcel.2020.05.005, PMID: 32502395

[ref9] Boutté Y. Jonsson K. McFarlane H. E. Johnson E. Gendre D. Swarup R.. (2013). ECHIDNA-mediated post-Golgi trafficking of auxin carriers for differential cell elongation. Proc. Natl. Acad. Sci. U. S. A. 110, 16259–16264. doi: 10.1073/pnas.1309057110, PMID: 24043780 PMC3791722

[ref10] Casler J. C. Glick B. S. (2020). A microscopy-based kinetic analysis of yeast vacuolar protein sorting. elife 9:e56844. doi: 10.7554/eLife.56844 32584255 PMC7338053

[ref11] Casler J. C. Johnson N. Krahn A. H. Pantazopoulou A. Day K. J. Glick B. S. (2022). Clathrin adaptors mediate two sequential pathways of intra-Golgi recycling. J. Cell Biol. 221:e202103199. doi: 10.1083/jcb.202103199, PMID: 34739034 PMC8576872

[ref12] Casler J. C. Papanikou E. Barrero J. J. Glick B. S. (2019). Maturation-driven transport and AP-1–dependent recycling of a secretory cargo in the Golgi. J. Cell Biol. 218, 1582–1601. doi: 10.1083/jcb.201807195, PMID: 30858194 PMC6504904

[ref13] Chow C. M. Neto H. Foucart C. Moore I. (2008). Rab-A2 and Rab-A3 GTPases define a *trans*-Golgi endosomal membrane domain in Arabidopsis that contributes substantially to the cell plate. Plant Cell 20, 101–123. doi: 10.1105/tpc.107.052001, PMID: 18239134 PMC2254926

[ref14] Collins N. C. Thordal-Christensen H. Lipka V. Bau S. Kombrink E. Qiu J. L.. (2003). SNARE-protein-mediated disease resistance at the plant cell wall. Nature 425, 973–977. doi: 10.1038/nature02076, PMID: 14586469

[ref15] Dacks J. B. Robinson M. S. (2017). Outerwear through the ages: evolutionary cell biology of vesicle coats. Curr. Opin. Cell Biol. 47, 108–116. doi: 10.1016/j.ceb.2017.04.001, PMID: 28622586

[ref16] Dahhan D. A. Reynolds G. D. Cárdenas J. J. Eeckhout D. Johnson A. Yperman K.. (2022). Proteomic characterization of isolated Arabidopsis clathrin-coated vesicles reveals evolutionarily conserved and plant-specific components. Plant Cell 34, 2150–2173. doi: 10.1093/plcell/koac071, PMID: 35218346 PMC9134090

[ref17] Day K. J. Casler J. C. Glick B. S. (2018). Budding yeast has a minimal endomembrane system. Dev. Cell 44:e4, 56–72. doi: 10.1016/j.devcel.2017.12.014, PMID: 29316441 PMC5765772

[ref18] Dettmer J. Hong-Hermesdorf A. Stierhof Y. D. Schumacher K. (2006). Vacuolar H+-ATPase activity is required for endocytic and secretory trafficking in Arabidopsis. Plant Cell 18, 715–730. doi: 10.1105/tpc.105.037978, PMID: 16461582 PMC1383645

[ref19] Di Rubbo S. Irani N. G. Kim S. Y. Xu Z. Y. Gadeyne A. Dejonghe W.. (2013). The clathrin adaptor complex AP-2 mediates endocytosis of brassinosteroid insensitive1 in Arabidopsis. Plant Cell 25, 2986–2997. doi: 10.1105/tpc.113.114058, PMID: 23975899 PMC3784593

[ref20] Ebine K. Inoue T. Ito J. Ito E. Uemura T. Goh T.. (2014). Plant vacuolar trafficking occurs through distinctly regulated pathways. Curr. Biol. 24, 1375–1382. doi: 10.1016/j.cub.2014.05.004, PMID: 24881878

[ref21] Ebine K. Okatani Y. Uemura T. Goh T. Shoda K. Niihama M.. (2008). A SNARE complex unique to seed plants is required for protein storage vacuole biogenesis and seed development of *Arabidopsis thaliana*. Plant Cell 20, 3006–3021. doi: 10.1105/tpc.107.057711, PMID: 18984676 PMC2613668

[ref22] El Kasmi F. Krause C. Hiller U. Stierhof Y. D. Mayer U. Conner L.. (2013). SNARE complexes of different composition jointly mediate membrane fusion in Arabidopsis cytokinesis. Mol. Biol. Cell 24, 1593–1601. doi: 10.1091/mbc.e13-02-0074, PMID: 23515225 PMC3655819

[ref23] Feng Q. N. Liang X. Li S. Zhang Y. (2018). The ADAPTOR PROTEIN-3 complex mediates pollen tube growth by coordinating vacuolar targeting and organization. Plant Physiol. 177, 216–225. doi: 10.1104/pp.17.01722, PMID: 29523712 PMC5933126

[ref24] Feng Q. N. Song S. J. Yu S. X. Wang J. G. Li S. Zhang Y. (2017). Adaptor protein-3-dependent vacuolar trafficking involves a subpopulation of COPII and HOPS tethering proteins. Plant Physiol. 174, 1609–1620. doi: 10.1104/pp.17.00584, PMID: 28559361 PMC5490925

[ref25] Fuji K. Shimada T. Takahashi H. Tamura K. Koumoto Y. Utsumi S.. (2007). Arabidopsis vacuolar sorting mutants (green fluorescent seed) can be identified efficiently by secretion of vacuole-targeted green fluorescent protein in their seeds. Plant Cell 19, 597–609. doi: 10.1105/tpc.106.045997, PMID: 17293568 PMC1867321

[ref26] Fuji K. Shirakawa M. Shimono Y. Kunieda T. Fukao Y. Koumoto Y.. (2016). The adaptor complex AP-4 regulates vacuolar protein sorting at the trans-Golgi network by interacting with vacuolar SORTING RECEPTOR1. Plant Physiol. 170, 211–219. doi: 10.1104/pp.15.00869, PMID: 26546666 PMC4704568

[ref27] Fujimoto M. Ueda T. (2012). Conserved and plant-unique mechanisms regulating plant post-Golgi traffic. Front. Plant Sci. 3:197. doi: 10.3389/fpls.2012.00197, PMID: 22973281 PMC3428585

[ref28] Geldner N. Anders N. Wolters H. Keicher J. Kornberger W. Muller P.. (2003). The Arabidopsis GNOM ARF-GEF mediates endosomal recycling, auxin transport, and auxin-dependent plant growth. Cell 112, 219–230. doi: 10.1016/S0092-8674(03)00003-5, PMID: 12553910

[ref29] Gendre D. McFarlane H. E. Johnson E. Mouille G. Sjödin A. Oh J.. (2013). Trans-Golgi network localized ECHIDNA/Ypt interacting protein complex is required for the secretion of cell wall polysaccharides in Arabidopsis. Plant Cell 25, 2633–2646. doi: 10.1105/tpc.113.112482, PMID: 23832588 PMC3753388

[ref30] Gendre D. Oh J. Boutté Y. Best J. G. Samuels L. Nilsson R.. (2011). Conserved Arabidopsis ECHIDNA protein mediates trans–Golgi-network trafficking and cell elongation. Proc. Natl. Acad. Sci. U. S. A. 108, 8048–8053. doi: 10.1073/pnas.1018371108, PMID: 21512130 PMC3093476

[ref31] Gershlick D. C. Lousa C. M. Foresti O. Lee A. J. Pereira E. A. da Silva L. L. P.. (2014). Golgi-dependent transport of vacuolar sorting receptors is regulated by COPII, AP1, and AP4 protein complexes in tobacco. Plant Cell 26, 1308–1329. doi: 10.1105/tpc.113.122226, PMID: 24642936 PMC4001386

[ref32] Glanc M. Fendrych M. Friml J. (2018). Mechanistic framework for cell-intrinsic re-establishment of PIN2 polarity after cell division. Nat. Plants. 4, 1082–1088. doi: 10.1038/s41477-018-0318-3, PMID: 30518833 PMC6394824

[ref33] Glick B. S. Nakano A. (2009). Membrane traffic within the Golgi apparatus. Annu. Rev. Cell Dev. Biol. 25, 113–132. doi: 10.1146/annurev.cellbio.24.110707.175421, PMID: 19575639 PMC2877624

[ref34] Grefen C. Chen Z. Honsbein A. Donald N. Hills A. Blatt M. R. (2010). A novel motif essential for SNARE interaction with the K^+^ channel KC1 and channel gating in Arabidopsis. Plant Cell 22, 3076–3092. doi: 10.1105/tpc.110.077768, PMID: 20884800 PMC2965544

[ref35] Griffiths G. Simons K. (1986). The trans Golgi network: sorting at the exit site of the Golgi complex. Science 234, 438–443. doi: 10.1126/science.2945253, PMID: 2945253

[ref36] Hachez C. Laloux T. Reinhardt H. Cavez D. Degand H. Grefen C.. (2014). Arabidopsis SNAREs SYP61 and SYP121 coordinate the trafficking of plasma membrane aquaporin PIP2;7 to modulate the cell membrane water permeability. Plant Cell 26, 3132–3147. doi: 10.1105/tpc.114.127159, PMID: 25082856 PMC4145137

[ref37] Harris N. Oparka K. J. (1983). Connections between dictyosomes, ER and GERL in cotyledons of mung bean (*Vigna radiata* L.). Protoplasma 114, 93–102. doi: 10.1007/BF01279872

[ref38] Heinze L. Freimuth N. Rößling A. K. Hahnke R. Riebschläger S. Fröhlich A.. (2020). EPSIN1 and MTV1 define functionally overlapping but molecularly distinct trans-Golgi network subdomains in Arabidopsis. Proc. Natl. Acad. Sci. U. S. A. 117, 25880–25889. doi: 10.1073/pnas.2004822117, PMID: 32989160 PMC7568309

[ref39] Hirst J. Barlow L. D. Francisco G. C. Sahlender D. A. Seaman M. N. J. Dacks J. B.. (2011). The fifth adaptor protein complex. PLoS Biol. 9:e1001170. doi: 10.1371/journal.pbio.1001170, PMID: 22022230 PMC3191125

[ref40] Holthuis J. C. M. Menon A. K. (2014). Lipid landscapes and pipelines in membrane homeostasis. Nature 510, 48–57. doi: 10.1038/nature13474, PMID: 24899304

[ref41] Honsbein A. Blatt M. R. Grefen C. (2011). A molecular framework for coupling cellular volume and osmotic solute transport control. J. Exp. Bot. 62, 2363–2370. doi: 10.1093/jxb/erq386, PMID: 21115662

[ref42] Honsbein A. Sokolovski S. Grefen C. Campanoni P. Pratelli R. Paneque M.. (2009). A tripartite SNARE-K^+^ channel complex mediates in channel-dependent K^+^ nutrition in Arabidopsis. Plant Cell 21, 2859–2877. doi: 10.1105/tpc.109.066118, PMID: 19794113 PMC2768940

[ref43] Huang Y. Ma T. Lau P. K. Wang J. Zhao T. Du S.. (2019). Visualization of protein sorting at the trans-Golgi network and endosomes through super-resolution imaging. Front. Cell Dev. Biol. 7:181. doi: 10.3389/fcell.2019.00181, PMID: 31552246 PMC6733968

[ref44] Ito Y. Boutté Y. (2020). Differentiation of trafficking pathways at Golgi entry core compartments and post-Golgi subdomains. Front. Plant Sci. 11:609516. doi: 10.3389/fpls.2020.609516, PMID: 33363561 PMC7752856

[ref45] Ito Y. Esnay N. Platre M. P. Wattelet-Boyer V. Noack L. C. Fougère L.. (2021). Sphingolipids mediate polar sorting of PIN2 through phosphoinositide consumption at the trans-Golgi network. Nat. Commun. 12:4267. doi: 10.1038/s41467-021-24548-0, PMID: 34257291 PMC8277843

[ref46] Ivanov R. Robinson D. G. (2020). EMAC, Retromer, and VSRs: do they connect? Protoplasma 257, 1725–1729. doi: 10.1007/s00709-020-01543-8, PMID: 32780164 PMC8286218

[ref47] Jackson C. L. Casanova J. E. (2000). Turning on ARF: the Sec7 family of guanine-nucleotide-exchange factors. Trends Cell Biol. 10, 60–67. doi: 10.1016/S0962-8924(99)01699-2, PMID: 10652516

[ref48] Jia P. F. Xue Y. Li H. J. Yang W. C. (2018). Golgi-localized LOT regulates *trans*-Golgi network biogenesis and pollen tube growth. Proc. Natl. Acad. Sci. U. S. A. 115, 12307–12312. doi: 10.1073/pnas.1809206115, PMID: 30413616 PMC6275481

[ref49] Jia P. F. Xue Y. Li H. J. Yang W. C. (2019). LOT regulates TGN biogenesis and Golgi structure in plants. Plant Signal. Behav. 14:e1573100. doi: 10.1080/15592324.2019.1573100, PMID: 30688137 PMC6422381

[ref50] Kang B. H. Nielsen E. Preuss M. L. Mastronarde D. Staehelin L. A. (2011). Electron tomography of RabA4b- and PI-4Kβ1-labeled *trans* Golgi network compartments in Arabidopsis. Traffic 12, 313–329. doi: 10.1111/j.1600-0854.2010.01146.x, PMID: 21134079

[ref51] Kim S. Y. Xu Z. Y. Song K. Kim D. H. Kang H. Reichardt I.. (2013). Adaptor protein complex 2-mediated endocytosis is crucial for male reproductive organ development in Arabidopsis. Plant Cell 25, 2970–2985. doi: 10.1105/tpc.113.114264, PMID: 23975898 PMC3784592

[ref52] Kitakura S. Vanneste S. Robert S. Löfke C. Teichmann T. Tanaka H.. (2011). Clathrin mediates endocytosis and polar distribution of PIN auxin transporters in Arabidopsis. Plant Cell 23, 1920–1931. doi: 10.1105/tpc.111.083030, PMID: 21551390 PMC3123958

[ref53] Koizumi K. Naramoto S. Sawa S. Yahara N. Ueda T. Nakano A.. (2005). VAN3 ARF-GAP-mediated vesicle transport is involved in leaf vascular network formation. Development 132, 1699–1711. doi: 10.1242/dev.01716, PMID: 15743878

[ref54] Kurokawa K. Okamoto M. Nakano A. (2014). Contact of cis-Golgi with ER exit sites executes cargo capture and delivery from the ER. Nat. Commun. 5:3653. doi: 10.1038/ncomms4653, PMID: 24728174 PMC3996532

[ref55] Kwon C. Neu C. Pajonk S. Yun H. S. Lipka U. Humphry M.. (2008). Co-option of a default secretory pathway for plant immune responses. Nature 451, 835–840. doi: 10.1038/nature06545, PMID: 18273019

[ref56] Lam S. K. Siu C. L. Hillmer S. Jang S. An G. Robinson D. G.. (2007). Rice SCAMP1 defines Clathrin-coated, trans-Golgi–located tubular-vesicular structures as an early endosome in tobacco BY-2 cells. Plant Cell 19, 296–319. doi: 10.1105/tpc.106.045708, PMID: 17209124 PMC1820953

[ref57] Law K. C. Chung K. K. Zhuang X. (2022). An update on coat protein complexes for vesicle formation in plant post-Golgi trafficking. Front. Plant Sci. 13:826007. doi: 10.3389/fpls.2022.826007, PMID: 35283904 PMC8905187

[ref58] Li R. Rodriguez-Furlan C. Wang J. van de Ven W. Gao T. Raikhel N. V.. (2017). Different endomembrane trafficking pathways establish apical and basal polarities. Plant Cell 29, 90–108. doi: 10.1105/tpc.16.00524, PMID: 28011692 PMC5304347

[ref59] Lin F. Krishnamoorthy P. Schubert V. Hause G. Heilmann M. Heilmann I. (2019). A dual role for cell plate-associated PI4Kβ in endocytosis and phragmoplast dynamics during plant somatic cytokinesis. EMBO J. 38:303. doi: 10.15252/embj.2018100303, PMID: 30617084 PMC6376452

[ref60] Lu C. Feng Z. Yuan F. Han G. Guo J. Chen M.. (2020). The SNARE protein LbSYP61 participates in salt secretion in Limonium bicolor. Environ. Exp. Bot. 176:104076. doi: 10.1016/j.envexpbot.2020.104076

[ref61] Marty F. (1978). Cytochemical studies on GERL, provacuoles, and vacuoles in root meristematic cells of Euphorbia. Proc. Natl. Acad. Sci. U. S. A. 75, 852–856. doi: 10.1073/pnas.75.2.852, PMID: 16592499 PMC411355

[ref62] Men S. Boutté Y. Ikeda Y. Li X. Palme K. Stierhof Y. D.. (2008). Sterol-dependent endocytosis mediates post-cytokinetic acquisition of PIN2 auxin efflux carrier polarity. Nat. Cell Biol. 10, 237–244. doi: 10.1038/ncb1686, PMID: 18223643

[ref63] Minamino N. Ueda T. (2019). RAB GTPases and their effectors in plant endosomal transport. Curr. Opin. Plant Biol. 52, 61–68. doi: 10.1016/j.pbi.2019.07.007, PMID: 31454706

[ref64] Müdsam C. Wollschläger P. Sauer N. Schneider S. (2018). Sorting of Arabidopsis NRAMP3 and NRAMP4 depends on adaptor protein complex AP4 and a dileucine-based motif. Traffic 19, 503–521. doi: 10.1111/tra.12567, PMID: 29573093

[ref65] Naramoto S. Sawa S. Koizumi K. Uemura T. Ueda T. Friml J.. (2009). Phosphoinositide-dependent regulation of VAN3 ARF-GAP localization and activity essential for vascular tissue continuity in plants. Development 136, 1529–1538. doi: 10.1242/dev.030098, PMID: 19363154

[ref66] Nishimura K. Matsunami E. Yoshida S. Kohata S. Yamauchi J. Jisaka M.. (2016). The tyrosine-sorting motif of the vacuolar sorting receptor VSR4 from *Arabidopsis thaliana*, which is involved in the interaction between VSR4 and AP1M2, μ1-adaptin type 2 of clathrin adaptor complex 1 subunits, participates in the post-Golgi sorting of VSR4. Biosci. Biotechnol. Biochem. 80, 694–705. doi: 10.1080/09168451.2015.1116925, PMID: 26745465

[ref67] Pang L. Ma Z. Zhang X. Huang Y. Li R. Miao Y.. (2022). The small GTPase RABA2a recruits SNARE proteins to regulate the secretory pathway in parallel with the exocyst complex in Arabidopsis. Mol. Plant 15, 398–418. doi: 10.1016/j.molp.2021.11.008, PMID: 34798312

[ref68] Park M. Song K. Reichardt I. Kim H. Mayer U. Stierhof Y. D.. (2013). Arabidopsis μ-adaptin subunit AP1M of adaptor protein complex 1 mediates late secretory and vacuolar traffic and is required for growth. Proc. Natl. Acad. Sci. U. S. A. 110, 10318–10323. doi: 10.1073/pnas.1300460110, PMID: 23733933 PMC3690844

[ref69] Peden A. A. Oorschot V. Hesser B. A. Austin C. D. Scheller R. H. Klumperman J. (2004). Localization of the AP-3 adaptor complex defines a novel endosomal exit site for lysosomal membrane proteins. J. Cell Biol. 164, 1065–1076. doi: 10.1083/jcb.200311064, PMID: 15051738 PMC2172074

[ref70] Pesacreta T. C. Lucas W. J. (1984). Plasma membrane coat and a coated vesicle-associated reticulum of membranes: their structure and possible interrelationship in *Chara corallina*. J. Cell Biol. 98, 1537–1545. doi: 10.1083/jcb.98.4.1537, PMID: 6425304 PMC2113203

[ref71] Preuss M. L. Schmitz A. J. Thole J. M. Bonner H. K. S. Otegui M. S. Nielsen E. (2006). A role for the RabA4b effector protein PI-4Kbeta1 in polarized expansion of root hair cells in *Arabidopsis thaliana*. J. Cell Biol. 172, 991–998. doi: 10.1083/jcb.200508116, PMID: 16567499 PMC2063757

[ref72] Ravikumar R. Kalbfuß N. Gendre D. Steiner A. Altmann M. Altmann S.. (2018). Independent yet overlapping pathways ensure the robustness and responsiveness of trans-Golgi network functions in Arabidopsis. Development 145, dev169201. doi: 10.1242/dev.169201, PMID: 30404777

[ref73] Renna L. Stefano G. Slabaugh E. Wormsbaecher C. Sulpizio A. Zienkiewicz K.. (2018). TGNap1 is required for microtubule-dependent homeostasis of a subpopulation of the plant trans-Golgi network. Nat. Commun. 9, 5313. doi: 10.1038/s41467-018-07662-4, PMID: 30552321 PMC6294250

[ref74] Richter S. Kientz M. Brumm S. Nielsen M. E. Park M. Gavidia R.. (2014). Delivery of endocytosed proteins to the cell–division plane requires change of pathway from recycling to secretion. elife 3:e02131. doi: 10.7554/eLife.02131, PMID: 24714496 PMC3979144

[ref75] Rodriguez-Furlan C. Campos R. Toth J. N. Van Norman J. M. (2022). Distinct mechanisms orchestrate the contra-polarity of IRK and KOIN, two LRR-receptor-kinases controlling root cell division. Nat. Commun. 13:235. doi: 10.1038/s41467-021-27913-1, PMID: 35017541 PMC8752632

[ref76] Rohn W. M. Rouillé Y. Waguri S. Hoflack B. (2000). Bi-directional trafficking between the *trans*-Golgi network and the endosomal/lysosomal system. J. Cell Sci. 113, 2093–2101. doi: 10.1242/jcs.113.12.2093, PMID: 10825282

[ref77] Saito C. Ueda T. (2009). Chapter 4: functions of RAB and SNARE proteins in plant life. Int. Rev. Cell Mol. Biol. 274, 183–233. doi: 10.1016/S1937-6448(08)02004-2, PMID: 19349038

[ref78] Sašek V. Janda M. Delage E. Puyaubert J. Guivarc H. A. López Maseda E.. (2014). Constitutive salicylic acid accumulation in pi4kIIIβ1β2 Arabidopsis plants stunts rosette but not root growth. New Phytol. 203, 805–816. doi: 10.1111/nph.12822, PMID: 24758581

[ref79] Sauer M. Delgadillo M. O. Zouhar J. Reynolds G. D. Pennington J. G. Jiang L.. (2013). MTV1 and MTV4 encode plant-specific ENTH and ARF GAP proteins that mediate clathrin-dependent trafficking of vacuolar cargo from the trans-Golgi network. Plant Cell 25, 2217–2235. doi: 10.1105/tpc.113.111724, PMID: 23771894 PMC3723622

[ref80] Shimada T. Kunieda T. Sumi S. Koumoto Y. Tamura K. Hatano K.. (2018). The AP-1 complex is required for proper mucilage formation in Arabidopsis Seeds. Plant Cell Physiol. 59, 2331–2338. doi: 10.1093/pcp/pcy158, PMID: 30099531

[ref81] Shimizu Y. Takagi J. Ito E. Ito Y. Ebine K. Komatsu Y.. (2021). Cargo sorting zones in the trans-Golgi network visualized by super-resolution confocal live imaging microscopy in plants. Nat. Commun. 12, 1901. doi: 10.1038/s41467-021-22267-0, PMID: 33772008 PMC7997971

[ref82] Singh M. K. Jürgens G. (2018). Specificity of plant membrane trafficking - ARFs, regulators and coat proteins. Semin. Cell Dev. Biol. 80, 85–93. doi: 10.1016/j.semcdb.2017.10.005, PMID: 29024759

[ref83] Singh M. K. Richter S. Beckmann H. Kientz M. Stierhof Y. D. Anders N.. (2018). A single class of ARF GTPase activated by several pathway-specific ARF-GEFs regulates essential membrane traffic in Arabidopsis. PLoS Genet. 14:e1007795. doi: 10.1371/journal.pgen.1007795, PMID: 30439956 PMC6264874

[ref84] Staehelin L. A. Giddings T. H. Jr. Kiss J. Z. Sack F. D. (1990). Macromolecular differentiation of Golgi stacks in root tips of Arabidopsis and Nicotiana seedlings as visualized in high pressure frozen and freeze-substituted samples. Protoplasma 157, 75–91. doi: 10.1007/BF01322640, PMID: 11537090

[ref85] Staehelin L. A. Kang B. H. (2008). Nanoscale architecture of endoplasmic reticulum export sites and of Golgi membranes as determined by electron tomography. Plant Physiol. 147, 1454–1468. doi: 10.1104/pp.108.120618, PMID: 18678738 PMC2492626

[ref86] Teh O. K. Shimono Y. Shirakawa M. Fukao Y. Tamura K. Shimada T.. (2013). The AP-1 μ adaptin is required for KNOLLE localization at the cell plate to mediate cytokinesis in Arabidopsis. Plant Cell Physiol. 54, 838–847. doi: 10.1093/pcp/pct048, PMID: 23543752

[ref87] Uemura T. Nakano R. T. Takagi J. Wang Y. Kramer K. Finkemeier I.. (2019). A Golgi-released subpopulation of the trans-Golgi network mediates protein secretion in Arabidopsis. Plant Physiol. 179, 519–532. doi: 10.1104/pp.18.01228, PMID: 30545905 PMC6426420

[ref88] Uemura T. Suda Y. Ueda T. Nakano A. (2014). Dynamic behavior of the trans-Golgi network in root tissues of Arabidopsis revealed by super-resolution live imaging. Plant Cell Physiol. 55, 694–703. doi: 10.1093/pcp/pcu010, PMID: 24443496

[ref89] Uemura T. Ueda T. (2014). Plant vacuolar trafficking driven by RAB and SNARE proteins. Curr. Opin. Plant Biol. 22, 116–121. doi: 10.1016/j.pbi.2014.10.002, PMID: 25460076

[ref90] Uemura T. Ueda T. Nakano A. (2012). The physiological role of SYP4 in the salinity and osmotic stress tolerances. Plant Signal. Behav. 7, 1118–1120. doi: 10.4161/psb.21307, PMID: 22899062 PMC3489641

[ref91] Viotti C. Bubeck J. Stierhof Y. D. Krebs M. Langhans M. van den Berg W.. (2010). Endocytic and secretory traffic in Arabidopsis merge in the trans-Golgi network/early endosome, an independent and highly dynamic organelle. Plant Cell 22, 1344–1357. doi: 10.1105/tpc.109.072637, PMID: 20435907 PMC2879741

[ref92] Waghmare S. Lileikyte E. Karnik R. Goodman J. K. Blatt M. R. Jones A. M. E. (2018). SNAREs SYP121 and SYP122 mediate the secretion of distinct cargo subsets. Plant Physiol. 178, 1679–1688. doi: 10.1104/pp.18.00832, PMID: 30348815 PMC6288737

[ref93] Wang X. Cai Y. Wang H. Zeng Y. Zhuang X. Li B.. (2014). trans-Golgi network-located AP1 gamma adaptins mediate dileucine motif-directed vacuolar targeting in Arabidopsis. Plant Cell 26, 4102–4118. doi: 10.1105/tpc.114.129759, PMID: 25351491 PMC4247576

[ref94] Wang P. Kang B. H. (2020). Correlative light and electron microscopy imaging of the plant trans-Golgi network. Methods Mol. Biol. 2177, 59–67. doi: 10.1007/978-1-0716-0767-1_6, PMID: 32632805

[ref95] Wang J. G. Li S. Zhao X. Y. Zhou L. Z. Huang G. Q. Feng C.. (2013). HAPLESS13, the Arabidopsis μ1 adaptin, is essential for protein sorting at the trans-Golgi network/early endosome. Plant Physiol. 162, 1897–1910. doi: 10.1104/pp.113.221051, PMID: 23766365 PMC3729769

[ref96] Wang Y. J. Wang J. Sun H. Q. Martinez M. Sun Y. X. Macia E.. (2003). Phosphatidylinositol 4 phosphate regulates targeting of clathrin adaptor AP-1 complexes to the Golgi. Cell 114, 299–310. doi: 10.1016/S0092-8674(03)00603-2, PMID: 12914695

[ref97] Wattelet-Boyer V. Brocard L. Jonsson K. Esnay N. Joubès J. Domergue F.. (2016). Enrichment of hydroxylated C24- and C26-acyl-chain sphingolipids mediates PIN2 apical sorting at trans-Golgi network subdomains. Nat. Commun. 7:12788. doi: 10.1038/ncomms12788, PMID: 27681606 PMC5056404

[ref98] Yamaoka S. Shimono Y. Shirakawa M. Fukao Y. Kawase T. Hatsugai N.. (2013). Identification and dynamics of Arabidopsis adaptor protein-2 complex and its involvement in floral organ development. Plant Cell 25, 2958–2969. doi: 10.1105/tpc.113.114082, PMID: 23975897 PMC3784591

[ref99] Yan X. Wang Y. Xu M. Dahhan D. A. Liu C. Zhang Y.. (2021). Cross-talk between clathrin-dependent post-Golgi trafficking and clathrin-mediated endocytosis in Arabidopsis root cells. Plant Cell 33, 3057–3075. doi: 10.1093/plcell/koab180, PMID: 34240193 PMC8462817

[ref100] Zhang C. Brown M. Q. van de Ven W. Zhang Z. M. Wu B. Young M. C.. (2016). Endosidin2 targets conserved exocyst complex subunit EXO70 to inhibit exocytosis. Proc. Natl. Acad. Sci. U. S. A. 113, E41–E50. doi: 10.1073/pnas.1521248112, PMID: 26607451 PMC4711834

[ref101] Zhang Z. Feechan A. Pedersen C. Newman M. A. Qiu J. L. Olesen K. L.. (2007). A SNARE-protein has opposing functions in penetration resistance and defence signalling pathways. Plant J. 49, 302–312. doi: 10.1111/j.1365-313X.2006.02961.x, PMID: 17241452

[ref102] Zhang L. Zhang H. Liu P. Hao H. Jin J. B. Lin J. (2011). Arabidopsis R-SNARE proteins VAMP721 and VAMP722 are required for cell plate formation. PLoS One 6:e26129. doi: 10.1371/journal.pone.0026129, PMID: 22022536 PMC3191180

[ref103] Zwiewka M. Feraru E. Möller B. Hwang I. Feraru M. I. Kleine-Vehn J.. (2011). The AP-3 adaptor complex is required for vacuolar function in Arabidopsis. Cell Res. 21, 1711–1722. doi: 10.1038/cr.2011.99, PMID: 21670741 PMC3357998

